# Secondary Metabolites from Marine-Derived *Bacillus*: A Comprehensive Review of Origins, Structures, and Bioactivities

**DOI:** 10.3390/md20090567

**Published:** 2022-09-06

**Authors:** Shaoyujia Xiao, Nan Chen, Zixue Chai, Mengdie Zhou, Chenghaotian Xiao, Shiqin Zhao, Xiliang Yang

**Affiliations:** Hubei Province Key Laboratory of Occupational Hazard Identification and Control, Department of Pharmacy, Institute of Infection, Immunology and Tumor Microenvironments, Medical College, Wuhan University of Science and Technology, Wuhan 430065, China

**Keywords:** marine microorganism, *Bacillus*, secondary metabolite, structural diversity, biological activity

## Abstract

The marine is a highly complex ecosystem including various microorganisms. *Bacillus* species is a predominant microbialflora widely distributed in marine ecosystems. This review aims to provide a systematic summary of the newly reported metabolites produced by marine-derived *Bacillus* species over recent years covering the literature from 2014 to 2021. It describes the structural diversity and biological activities of the reported compounds. Herein, a total of 87 newly reported metabolites are included in this article, among which 49 compounds originated from marine sediments, indicating that marine sediments are majority sources of productive strains of *Bacillus* species Therefore, marine-derived *Bacillus* species are a potentially promising source for the discovery of new metabolites.

## 1. Introduction

The ocean is a highly complex ecosystem, a rich and underdeveloped treasure house containing a wide variety of biological resources including aquatic species and various microorganisms [1,2,3]. Natural products, especially small molecules isolated from biological sources, have long been regarded for their huge potential in human medicine and are still gaining traction [4]. Marine microorganisms produce many undiscovered molecules with unprecedented structures and pharmacological activities in an extreme living environment [2]. Therefore, it is commonly recognized that marine microbes constitute a promising source of novel metabolites with considerable therapeutically potential for new drug screening and development [3,5,6,7,8].

*Bacillus* species is a predominant microbialflora widely distributed in marine ecosystems [9,10]. *Bacillus* species can grow rapidly and tolerate extremely adverse environmental conditions such as extreme ambient temperature, salinity and pH, high pressure and nutrient deficiency [11]. *B. subtilis* can adopt several responses when faced with the depletion of essential nutrients, including motility, secretion of extracellular enzymes, genetic transformation, antibiotic production, and finally sporulation [12]. The genus *Bacillus* is a prolific producer of bioactive metabolites, including more than 350 kinds of rod-shaped and Gram-positive bacteria [13]. Thereinto, *B. subtilis*, *B. licheniformis* and *B. amyloliquefaciens* possess potential value as therapeutic agent candidates on account of their ability to produce bioactive secondary metabolites [14,15,16,17].

In recent years, a variety of secondary metabolites of marine *Bacillus* species have been studied, including lipopeptides [18], polyketides [19], non-ribosomal peptides [20], macrolides [16,21], and glycopeptides [9]. Several natural products were isolated from the marine organisms for drug development by traditional means of bioactivity-guided methods and chemical structure elucidation. More precise analytical methods, such as LC-MS and NMR spectroscopy guided metabolic profiling and dereplication of a crude extract, also promoted the emergence of new secondary metabolites [22,23,24]. Some novel technologies, such as improved genome mining methods, have propelled natural products in the field of drug discovery [25]. The marine hosts a huge variety of organisms adapted to the specific environment, which should result in the production of a wide range of unique biomolecules [7]. The production of secondary metabolites is normally associated with the bacterium’s response to a growth-limiting environment; hence, exploring the high diversity of marine environments may uncover multiple compounds with unique structures and biological activities [26]. For example, most of this industrial *Bacillus pumilus* group (*Bp* group) have been isolated from terrestrial ecosystems at present. By contrast, members of the *Bp* group are ubiquitous and diverse in marine environments, but less explored [27].

According to studies, these compounds have a wide range of biological activities, viz., antimicrobial [28,29], anticancer [30,31,32], antivirus [33], antifungal, promotion of plant growth [34], immunosuppressive, antituberculosis, antimycoplasmic and exceptional surfactant [35], indicating their promising medicinal, agricultural and industrial potential. Over the past decades, *Firmicutes phylum* were found to be the marine-derived bacteria producers of the most antimicrobial activity, *Bacillus* strains specifically [26]. Sequel of genomic analyses demonstrated the prospect of marine *Bacillus* species producing wide-ranging polyketide classes of antibiotic agents, such as macrocyclic lactones, bacillaene, macrolactins, and difficidins [36,37]. Polyketide, as conspicuous bactericidal agents in the human health area, was reported to produce by multifunctional microbial polyketide synthase (pks) complex [38]. Difficidins are a polyketide class of polyenes, which were reported to biosynthesize by type I *pks* encrypted in the *dif* operon and were recognized to hinder bacterial pathogens [35]. Moreover, polyketides are the most structurally diverse and pharmacologically relevant natural products with low toxicity and high efficacy, many of which exhibit cytotoxic effects on cancer cells.

While numerous articles address *Bacillus* species and their secondary metabolites in general or the renewal biological functionalities, an overview of recently discovered compounds produced by marine *Bacillus* is missing. Only one review of *Bacillus* species was reported, by Mondol MA et al. in 2013 [39]. Therefore, we delved into the literature from the beginning of 2014 to the end of 2021. To the best of our knowledge, a summary of such literature is provided along with the novel chemical structures, diverse producing strains, environmental sources, pharmacological activities, employed experimental and putative biosynthetic pathways as well.

**Figure 1:**
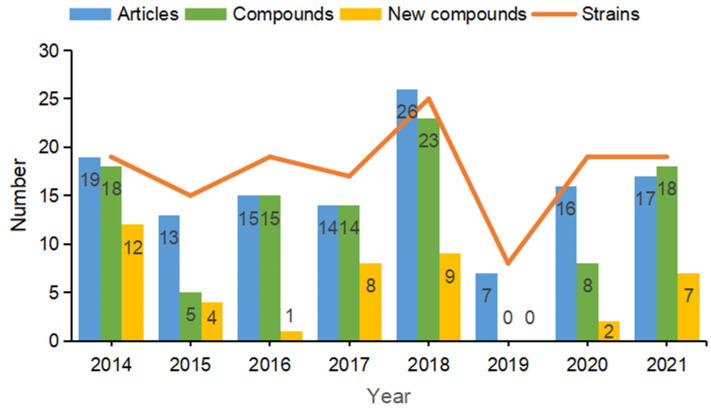
The numbers of articles, compounds, and strains reported by marine-derived *Bacillus* species from 2014–2021.

The literature retrieval was performed using a previously reported method [40,41]. All original articles were collected in the present review by searching various databases, including PubMed, Web of Science, China National Knowledge Infrastructure (CNKI) between 2014 and 2021. The search strategy was as follows: “Title: (*Bacillus*); Refined by: Topic (marine) and Document types (article); Timespan: 2014–2021”. A total of 127 hits were made. Only papers with reports about isolation of secondary metabolites are considered in this review. Other reports, including genome sequencing, industry, and ecological environmental studies, are touched upon but are not described comprehensively. It should be noted that this review was initially planned for the end of 2021. The studies published or being submitted in the current year might not be indexed in the PubMed in a timely manner; thus, the time span of literature search is from 2014 till 2021. During this period, a total of 87 secondary metabolites were isolated and characterized from marine-derived *Bacillus* species, which were reported in 33 original articles and account for 25.98% of these reported publications. The numbers of articles, strains and compounds were collected and collated according to the year they were published. Results indicated that the numbers of strains and compounds reported to be produced by marine-derived *Bacillus* species have been fluctuating over the past eight years (between 2014 and 2021). In particular, data showed that there were no articles reporting compounds isolated from marine-derived *Bacillus* species in 2019 (Figure 1).

## 2. Structural Diversity

This review summarizes the newly reported secondary metabolites (**1**–**87**) produced by marine-derived *Bacillus* species from 2014 till 2021. These secondary metabolites could be classified into six major categories: cyclic lipopeptides (**1**–**19**); diketopiperazines (**20**–**27**); linear lipopeptides (**28**–**38**); polyketides (**39**–**44**); nonribosomal peptides (**45**–**57**); macrolactins (**58**–**72**); and other compounds (**73**–**87**) based on their structural patterns.

### 2.1. Cyclic Lipopeptides

**Figure 2:**
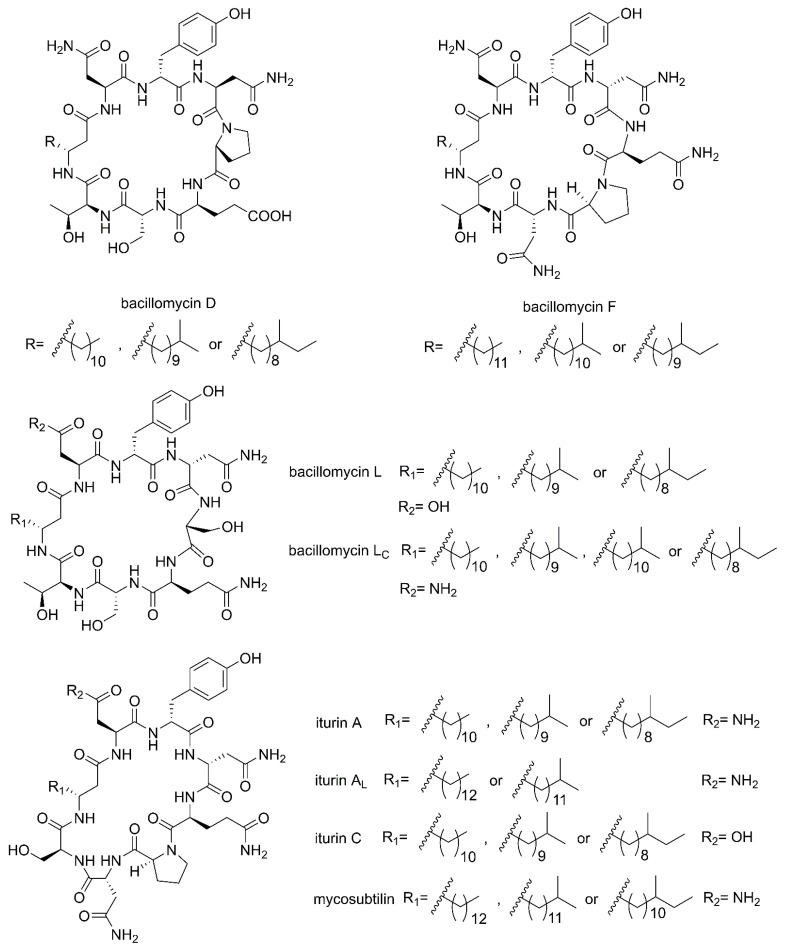
The structures of iturin family members (bacillomycin D, F, L, Lc, iturin A, AL, C and mycosubtilin).

**Figure 3:**
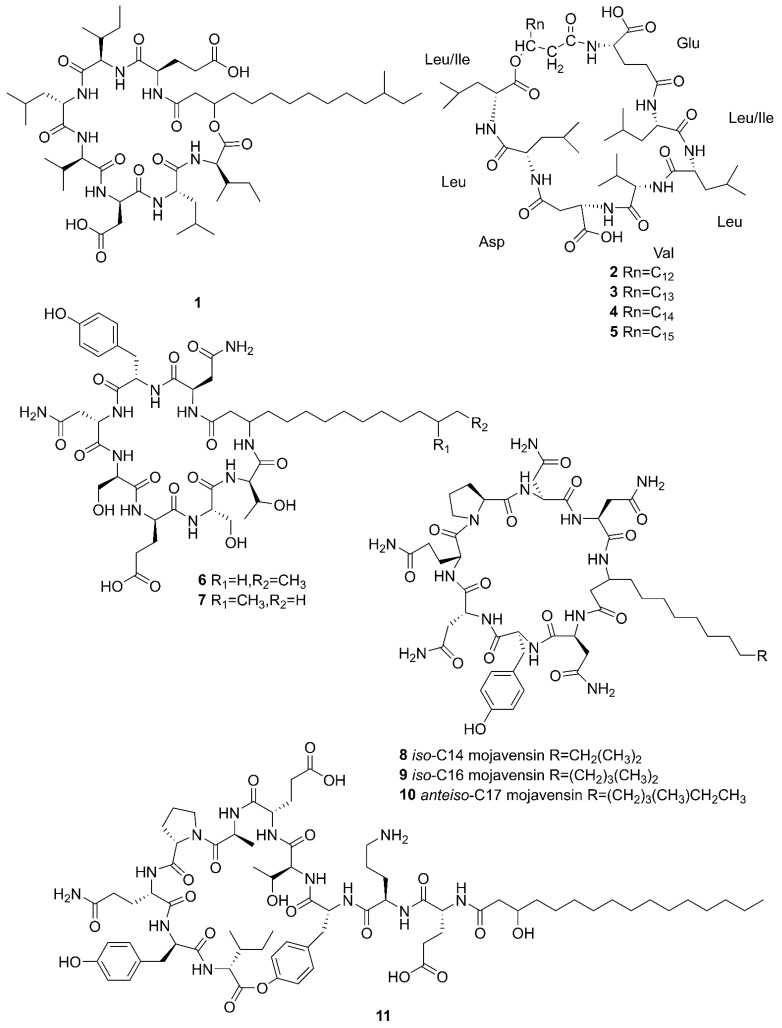

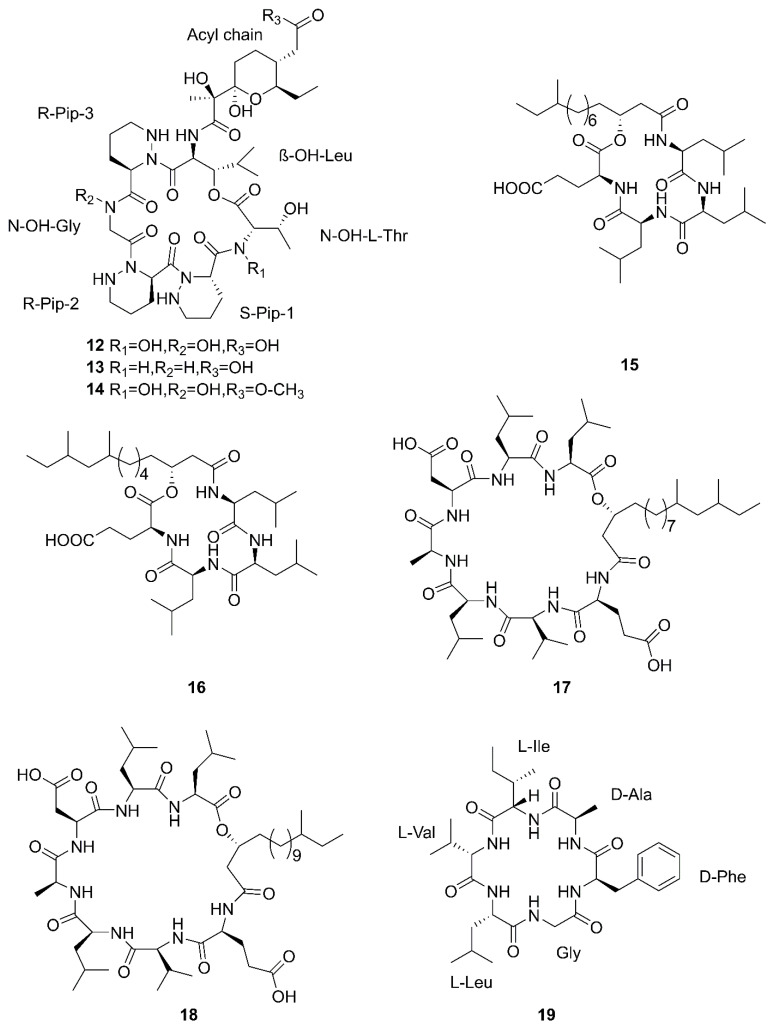
Cyclic lipopeptides produced by marine-derived *Bacillus* species.

Cyclic lipopeptides (CLPs) are common secondary metabolites isolated from marine-derived *Bacillus*. The CLPs are a class of metabolites with structural diversity produced by multifarious bacterial genera [42]. There are three families of CLPs being of particular importance, namely surfactins, iturins and plipastatins, all consisting of a short cyclic oligopeptide linked to the tail of a fatty acid [14]. Surfactin sequences comprise of seven amino acids and a *β*-hydroxy fatty acid chain containing 12–16 carbons [43]. The iturin family sequences are composed of heptapeptides and a *β*-amino fatty acid chain of 14–17 carbon atoms, which consists of bacillomycin D, F, L, Lc, iturin A, A_L_, C and mycosubtilin (Figure 2) [44]. The plipastatin family comprise of ten amino acids and a *β*-hydroxy fatty acid containing 14–18 carbon atoms [45]. Figure 3 lists the structures of cyclic lipopeptides produced by marine-derived *Bacillus* species.

Compounds **1–5** belong to surfactin family. A new CLP surfactin named *anteiso*-C_15_ Ile_2,7_ surfactin (**1**) was isolated from *B*. *velezensis* SH-B74 in the China Center for Type Culture Collection (CCTCC), which collected from the marine sediments, comprising of an *anteiso*-C_15_ type saturated fatty acid chain, and a peptidic backbone of L-Glu_1_, L-Ile_2_, D-Leu_3_, L-Val_4_, L-Asp_5_, D-Leu_6_, L-Ile_7_ [46]. Rn-Glu^1^-Leu/Ile^2^-Leu^3^-Val^4^-Asp^5^-Leu^6^-Leu/Ile^7^ (**2–5**) belonging to surfactin homolog were isolated from *B*. *licheniformis* MB01 collected from sediments in the Bohai Sea, China [47]. Compounds **6–10** belong to the iturin family. A novel lipopeptide antibiotic bacillopeptin named bacillopeptin B_1_ (**6**) and a known compound, bacillopeptin B (**7**) were detected in the fermentation broth of a marine sediment-derived *B. amyloliquefaciens* SH-B74 collected from sediments in the South China Sea. More precisely, compound **6** as a member of bacillopeptin family has the same amino-acid sequence and the same molecular weight as compound **7**, but has a different fatty-acid residue [22]. Compounds **8–10** were characterized as cyclic lipopeptides with saturated *β*-amino fatty acid chain residues, *iso*-C14 mojavensin, *iso*-C16 mojavensin, and *anteiso*-C17 mojavensin, all of which were produced by a marine-derived *B. mojavensis* B0621A obtained from the mantle of a pearl oyster *Pinctada martensii* in the South China Sea [48]. In addition, plipastatin A1 (**11**), belonging to the plipastatin family, was obtained by solidphase extraction and reversed-phase high-performance liquid chromatograph (RP-HPLC) from the fermentation broth of a marine sediment-derived *B. amyloliquefaciens* SH-B74 in the CCTCC [49]. A new cyclic hexapeptide with three piperazic acids (N-OH-Thr, N-OH-Gly, *β*-OH-Leu) named dentigerumycin E (**12**) and two reported derivatives, 2-N, 16-N-deoxydenteigerumycin E (**13**) and dentigerumycin E methyl ester (**14**), were isolated from coculture of marine *Streptomyces* and *Bacillus* strains collected together from the intertidal mudflat in Wando, Republic of Korea. It is worth mentioning that only compound **12** showed antiproliferative and antimetastatic activities against human cancer cells, suggesting that 2-N-OH, 16-N-OH, and 37-OH (carboxylic acid) are essential for the activities [25]. Two novel cyclic lipopeptides, bacilotetrin A (**15**) and bacilotetrin B (**16**), possessing three leucines and one glutamic residue cyclized with a lipophilic 3-hydroxyl fatty acid, were isolated from *B. subtilis* 109GGC020 in the sediments from the Gageocho of southern reef, Republic of Korea [12]. Additionally, gageopeptins A (**17**) and B (**18**), two novel cyclic lipopeptides, were isolated from the same strain in the same sediments as above [50]. A new cyclic hexapeptide named bacicyclin (**19**) was purified from *Bacillus* sp. BC028 associated with the blue mussel *Mytilus edulis* collected from the western shore of the Baltic Sea in Germany [51].

### 2.2. Diketopiperazines

**Figure 4:**
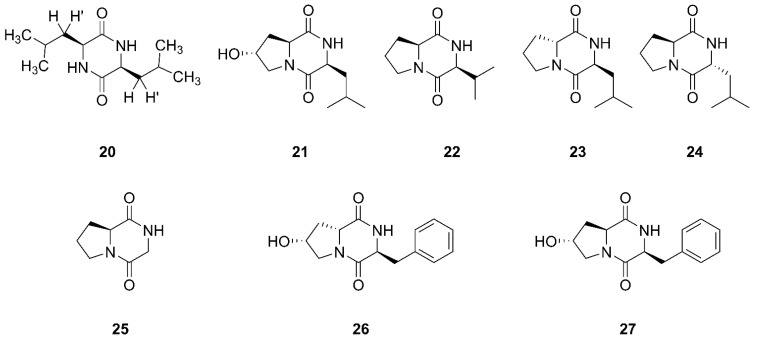
Diketopiperazines produced by marine-derived *Bacillus* species.

Cyclicpeptide diketopiperazines consist of residues of two amino acids and mevalonic acid [52]. Figure 4 lists the structures of diketopiperazines that were produced by marine-derived *Bacillus* species.

Compound **20** was established as a diketopiperazine (3S, 6S)-3,6-diisobutylpiperazine-2,5-dione, which was isolated from the ethyl acetate extract of the culture broth of *Bacillus* sp. SPB7. This strain SPB7 was obtained from marine sponge *Spongia officinalis* collected from the Palk Bay of Bengal, India. In particular, this is the first time that compound **20** had been isolated from a sponge-associated microbe [53]. Finally, seven cyclic dipeptides compounds **21**–**27** were identified and characterized as cyclo (L-leu-trans-8-hydroxy-L-pro), cyclo (L-val-L-pro), cyclo (D-pro-L-leu), cyclo (L-pro-D-leu), cyclo (gly-L-pro), cyclo (L-phe-cis-8-hydroxy-D-pro), and cyclo (L-phe-trans-8-hydroxy-L-pro), respectively, which were isolated from *Bacillus* sp. UST050418-715 collected from sponge in the sea near St. Juan Island, Washington, USA [54]. Compound **25** was also found from sponge-endosymbiotic *Bacillus* species collected from Agatti island located in the Arabian Sea, in the Laksha Archipelago of India [55].

### 2.3. Linear Lipopeptides

**Figure 5:**
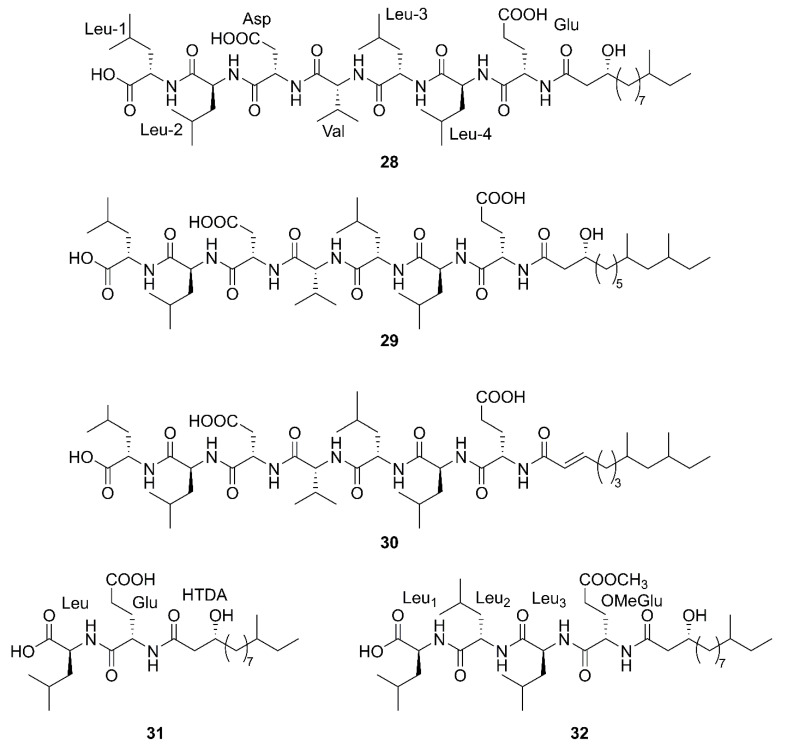

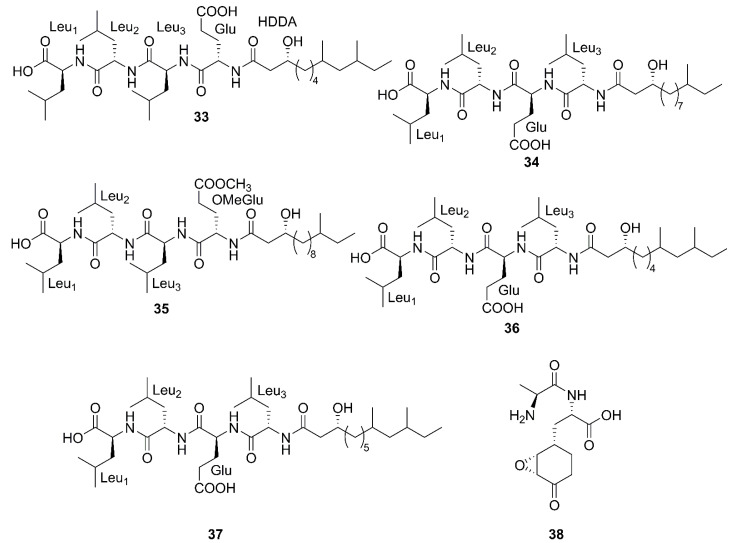
Linear lipopeptides produced by marine-derived *Bacillus* species.

Linear lipopeptide is a kind of lipopeptide, in which amino acids are connected in turn into linear, unconnected head and tail and no cyclic structure. Fatty acids are connected to α-amino groups or other hydroxyl groups at the N-terminal of the peptide chain [56]. Figure 5 lists the structures of linear lipopeptides that were produced by marine-derived *Bacillus* species.

Three newfound linear lipopeptides named gageostatins A (**28**), B (**29**) and C (**30**), comprising of hepta-peptides and new 3-*β*-hydroxy fatty acids yielded by a marine-derived bacterium *B. subtilis* from the culture broth [35]. Furthermore, three novel linear lipopeptides possessing di- and tetrapeptides and a new fatty acid, gageotetrins A (**31**), B (**32**) and C (**33**), were isolated from a marine *B. subtilis* [57]. Four unreported lipopeptides, gageopeptides A (**34**), B (**35**), C (**36**), and D (**37**) were isolated and identified from a marine-derived bacterium *B. subtilis*, which consisted of tetrapeptides and 3-*β*-hydroxy fatty acids [58]. The fatty acid of **28**, **31**, **32** and **34** was identical and determined as a 3-*β*-hydroxy-11-methyltridecanoic acid. Likewise, compounds **29** and **37** both possessed the same fatty acid, 3-*β*-hydroxy-9,11-dimethyltridecanoic acid. Moreover, the fatty acid unit of **33** and **36** was 3-*β*-hydroxy-8,10-dimethyldodecanoic acid. In particular, the absolute stereochemistry at C-3 of the fatty acids of linear lipopeptides **28**–**37** is *R* configuration except **30**. Additionally, the configuration of the amino acid residues in **28**–**37** was found to be *L*-form, while Val in **28**–**30** was *D*-form. Besides, bacilysin (**38**), another identified dipeptide, was isolated from seaweed-associated *B. amyloliquefaciens* MTCC 10456 in Microbial Type Culture Collection and Gene Bank (MTCC) of Chandigarh in India. Notably, this is the first report on the co-production and isolation of anti- *Malassezia* spp. chemicals from marine *Bacillus* species [44]. In conclusion, all linear Lipopeptide mentioned above were obtained from the Gageocho in the southern reef (Republic of Korea) except **38**.

### 2.4. Nonribosomal Peptides

**Figure 6:**
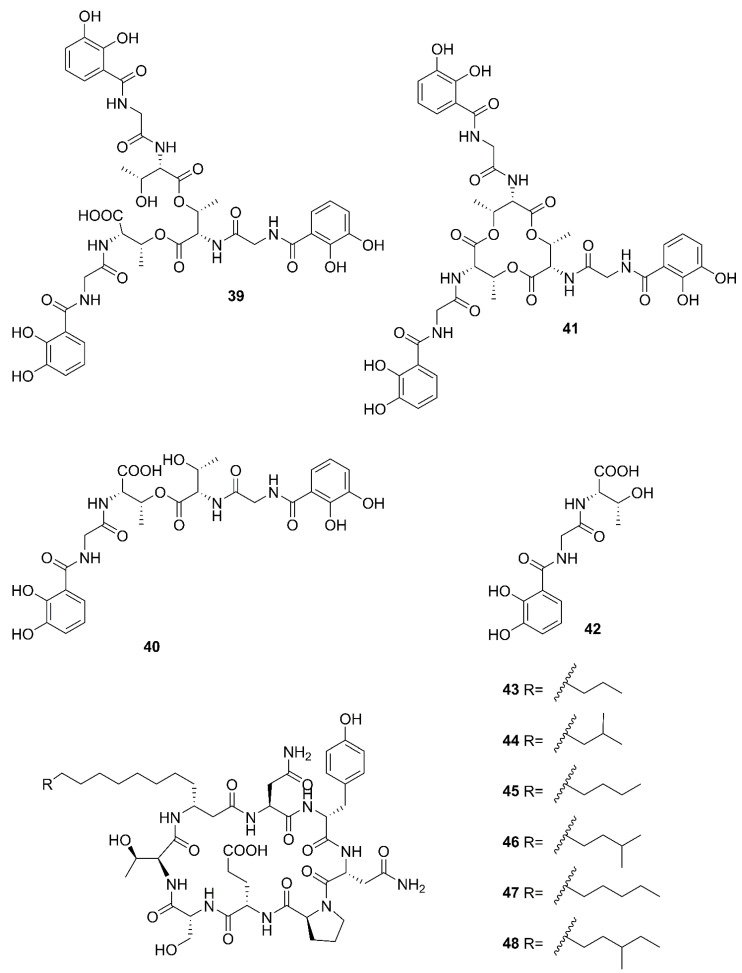

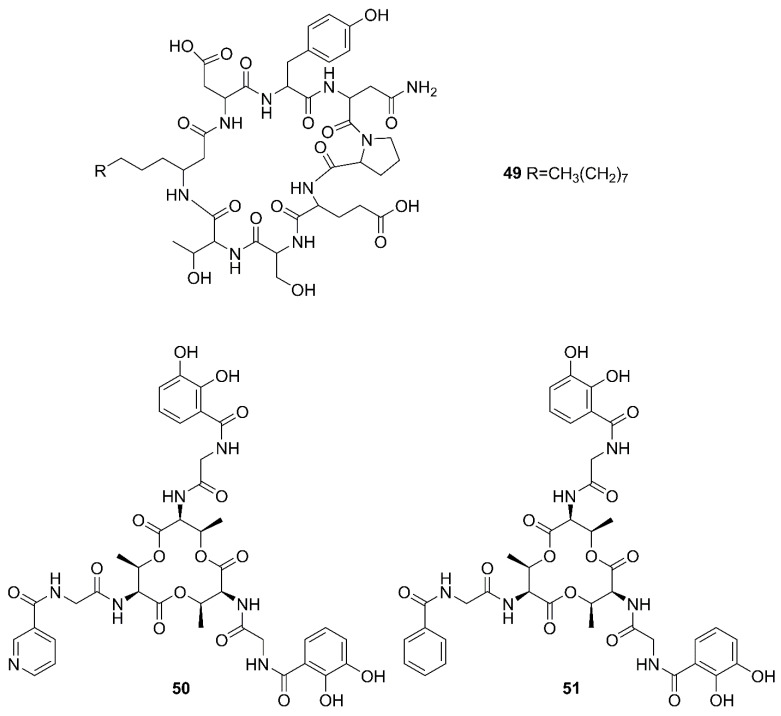
Nonribosomal peptides produced by marine-derived *Bacillus* species.

Nonribosomal peptides (NRPs) are large enzyme complexes with a modular structure responsible for binding a particular amino acid. NRPSs of *Bacillus* are synthesized by large multimodular nonribosomal peptide-synthetase (NRPS) through prolonging the active monomers of amino acid building blocks [59]. Figure 6 lists the structures of nonribosomal peptides produced by marine-derived *Bacillus* species.

Two unreported compounds, bacillibactin B (**39**) and bacillibactin C (**40**), along with the known compounds Bacillibactin (**41**) and S_VK21_ (**42**), were discovered from *Bacillus* sp. named PKU-MA00093 from sponges, corals and sediments in the South China Sea. Additionally, compounds **43**–**48** were characterized as bacillomycin D, *iso*-C15 bacillomycin D, C15 bacillomycin D, *iso*-C16 bacillomycin D, C16 bacillomycin D, and *anteiso*-C17 bacillomycin D, respectively. They were isolated from *Bacillus* sp. PKU-MA00092 collected from sponges, corals and sediments in the South China Sea. Notably, this was the first time to report the structures of **45** and **47** with fully specified ^1^H NMR and ^13^C NMR data; their structures are highly similar except for the fatty acid moieties [60]. Compounds **45** and **47** in company with C14 bacillomycin D (**49**), were obtained from seaweed-associated *B. amyloliquefaciens* MTCC 10456 collected from seaweed in the MTCC, of Chandigarh, India [44]. Moreover, compounds **45** and **49** were also isolated from the methanol extract harvested from marine-derived *B. megaterium* CGMCC7086 obtained from the intestines of marine fish in the Yellow Sea of East China by two-step ultrafiltration and liquid chromatography-electronic spray ionization-tandem mass spectrometry (LC-ESI-MS/MS). Using a highly-efficient separation technique and identification method, more than 40 lipopeptides variants were identified from a *Bacillus* strain [23]. Besides, compound **47** was isolated from *B. subtilis* B38 strain [61]. Two unique bacillibactins, bacillibactins E (**50**) and F (**51**) were the first bacterial siderophores containing nicotinic and benzoic acid moieties isolated from a marine sponge *Cinachyrella apion* associated *Bacillus* sp. WMMC1349 collected from the west shore of Ramrod Key in Florida [24].

### 2.5. Polyketides

**Figure 7:**
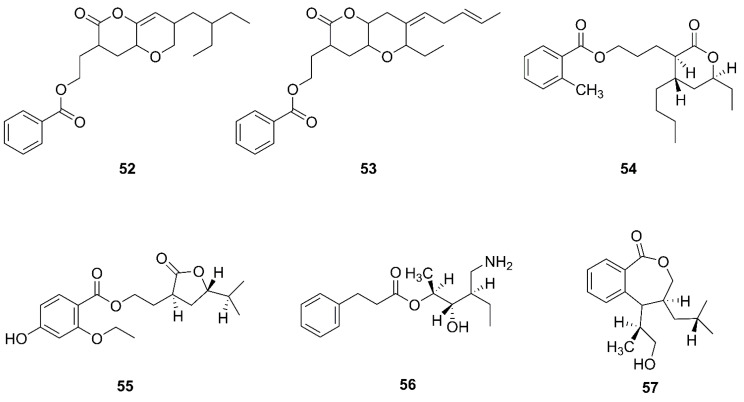
Polyketides produced by marine-derived *Bacillus* species.

Polyketides are a class of extremely large secondary metabolites assembled from simple acyl-coA compounds [62]. *Bacillus* species of marine origin was a potential source of bioactive compounds of polyketides and bacteriocins with significant antimicrobial activity against human pathogens [63]. Figure 7 lists the structures of polyketides that were produced by marine-derived *Bacillus* species.

Two novel compounds, O-heterocycle pyrans, 2-(7-(2-Ethylbutyl)-2,3,4,4a,6,7-hexahydro-2-oxopyrano-[3,2b]-pyran-3-yl)-ethyl benzoate (**52**) and 2-((4Z)-2-ethyl-octahydro-6-oxo-3-((E)-pent-3-enylidene)-pyrano-[3,2b]-pyran-7-yl)-ethyl benzoate (**53**) were obtained by repeated chromatography from the heterotrophic bacterium *B. subtilis* MTCC 10407 associated with brown seaweed *Sargassum myriocystum* on the southeast coast of India [64]. Additionally, 11-(15-butyl-13-ethyl-tetrahydro-12-oxo-2H-pyran-13-yl) propyl-2-methylbenzoate (**54**), 9-(tetrahydro-2-isopropyl-11-oxofuran-10-yl)-ethyl-4-ethoxy-2-hydroxybenzoate (**55**), 12-(aminomethyl)-11-hydroxyhexanyl-10-phenylpropanoate (**56**), and 7-(14-hydroxypropan-13-yl)-8-isobutyl-7, and 8 dihydrobenzo[c]oxepin-1(3H)-one (**57**) were isolated from a heterotrophic marine bacterium *B. amyloliquefaciens.* This strain was isolated from the brown seaweed *Padina gymnospora* collected from the intertidal zone of the Mannar Gulf in Peninsular India [63].

### 2.6. Macrolactins

**Figure 8:**
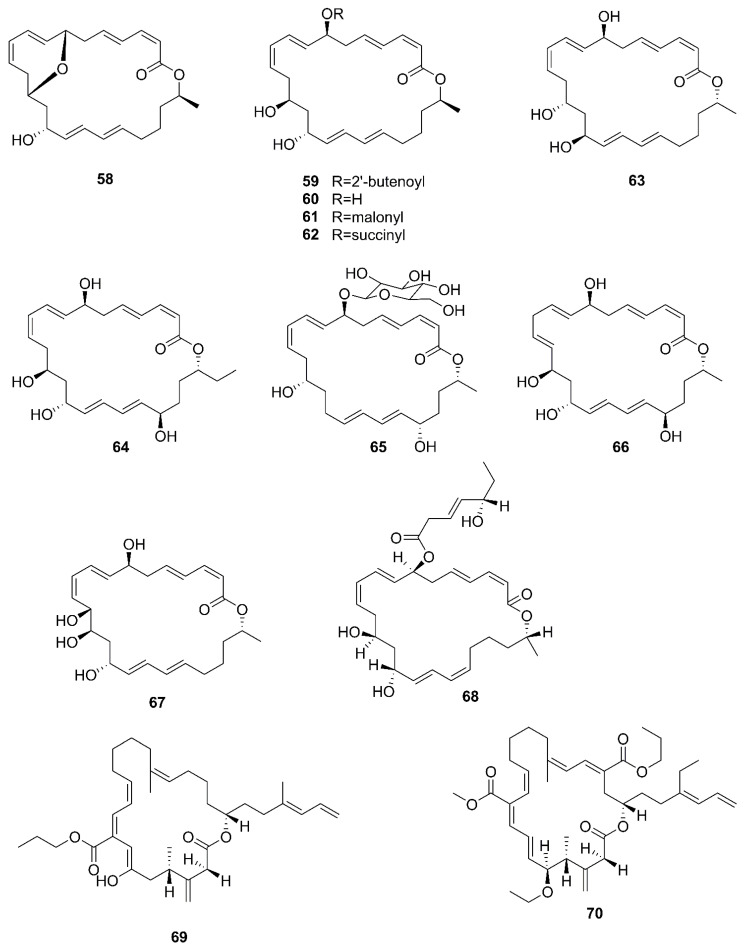

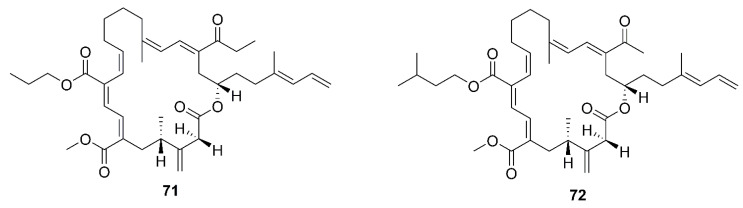
Macrolactins produced by marine-derived *Bacillus* species.

Marine *Bacillus* species produce abundant polyketide classes of antibiotic agents, such as macrolactins, difficidins, and bacillaenes [13,36,65]. Diffcidin is a highly unsaturated macrocyclic polyene with a 22-membered carbon skeleton and a phosphate moiety, which is rarely found in secondary metabolites of *Bacillus* species. Bacillaene is a linear structure consisting of a conjugated hexaene. Carbon skeleton of most macrolactins contains three diene groups attached to the carbon backbone of a 24-membered lactone ring [36]. Figure 8 lists the structures of macrolactins produced by marine-derived *Bacillus* species.

A new macrolactin derivative, 7,13-epoxyl-macrolactin A (**58**), along with four known macrolactins, 7-O-2′E-butenoyl macrolactin A (**59**), Macrolactin A (**60**), 7-O-malonyl macrolactin A (**61**), and 7-O-succinyl macrolactin A (**62**) were isolated from bacteria *B. subtilis* B5. It is worth emphasizing that this strain was extracted from deep-sea sediments at depths of 3000 m in the Pacific Ocean [66]. Compounds **60** and **62** were also isolated from seaweed-associated *B. amyloliquefaciens* MTCC 10456 in the MTCC of Chandigarh, India; this is the first report on the co-production and isolation of anti-*Malassezia* spp. compounds from marine *Bacillus* species [44]. In particular, the major difference between compounds **58** and **59**–**62** is in the epoxy ring. Compound **58** displayed a potent inhibitory effect on the expression of interleukin-1*β* (IL-1*β*), interleukin-6 (IL-6) and inducible nitric oxide synthase (iNOS), due to the existence of the epoxy ring [66]. Five novel 24-membered macrolactins named bamemacrolactins A (**63**), B (**64**), C (**65**), D (**66**) and E (**67**) were produced by *B. siamensis,* which was isolated from the gorgonian coral *Anthogorgia caerulea* gathered from Beihai city (Guangxi, China) [67]. The 7-O-methyl-5′-hydroxy-3′-heptenoate−macrolactin (**68**), a new macrolactin compound, was obtained from *B. subtilis* MTCC10403 associated with seaweed *Anthophycus longifolius* collected from the Gulf of Mannar of Peninsular India [68]. Compounds **69**–**72** were characterized as four homologous difficidin-type 21-membered macrocyclic lactone, isolated from a heterotrophoic *B. amyloliquefaciens* MTCC12713 associated with an intertidal macroalga *Kappaphycus alverezii* collected from the Gulf of Mannar in Peninsular India. In addition, they were established as 18,19-dihydro-6-hydroxy-8-propyl carboxylate difficidin, 5-ethoxy-28-methyl-(9-methyl-19propyl dicarboxylate) difficidin, (6-methyl-9-propyl dicarboxylate)-19-propanone difficidin, and 20-acetyl-(6-methyl-9-isopentyl dicarboxylate) difficidin, respectively [13].

### 2.7. Other Compounds

**Figure 9:**
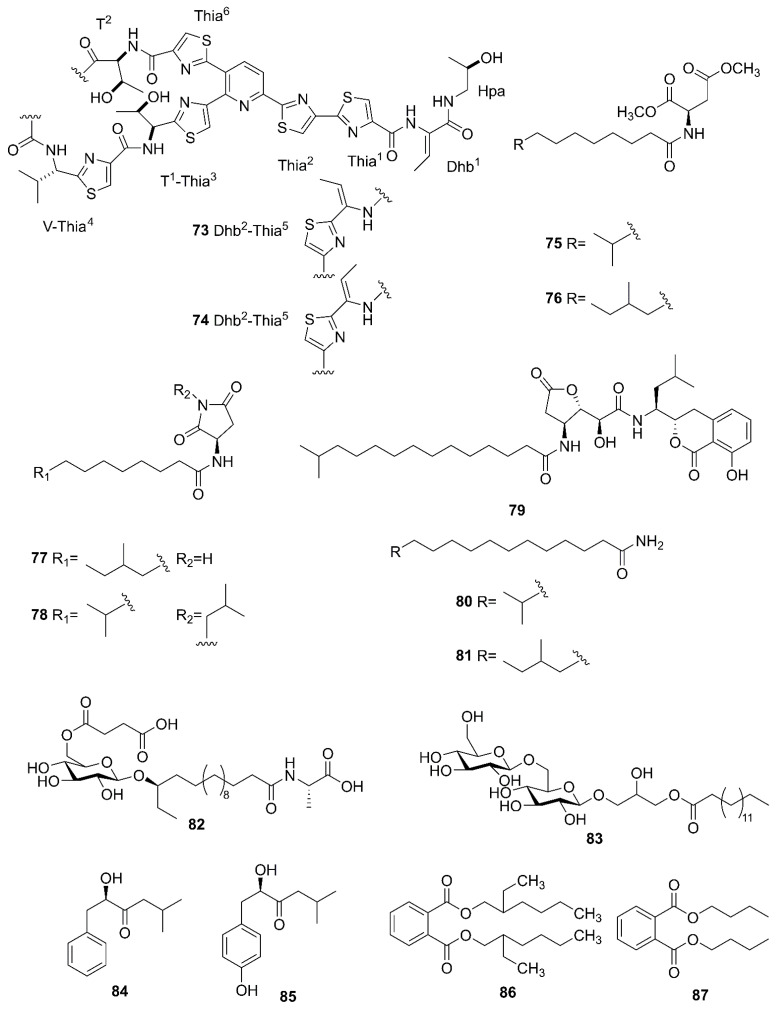
Other compounds produced by marine-derived *Bacillus* species.

Figure 9 lists the other compounds produced by marine-derived *Bacillus* species. A novel thiopeptide named micrococcin P3 (**73**) and a known compound named micrococcin P1 (**74**) were isolated from the fermentation broth of *B. stratosphericus* [69]. Five new bacillamidins A (**75**), B (**76**), C (**77**), D (**78**) and E (**79**), along with two known synthetic analogs, bacillamidins F (**80**) and G (**81**), were isolated from the marine-derived *B. pumilus* strain RJA1515. This strain was extracted from deep-sea sediments at depths of 84 m collected in Bamfield in British Columbia [70]. Ieodoglucomide C (**82**) and ieodoglycolipid (**83**), two new glycolipids, were produced by the marine-derived *B. licheniformis* 09IDYM23 which was isolated from sediments at a depth 20 m collected at Ieodoin the southern reef of the Republic of Korea, both of which were obtained from the fermentation of this strain [71]. According to bioactivity-guided strategy, (-)-sattabacin (**84**) and (-)-4-hydroxysattabacin (**85**) were firstly discovered from *Bacillus* sp. (SCO-147) collected from marine sediments in Suncheon Bay of Korea [72]. Marine-derived *B. subtilis* AD35, gathered from marine water and sediment at the Alexandria sea shore in Egypt, could yield a previously reported but firstly isolated compound, Di-(2-ethylhexyl) phthalate (DEHP) (**86**) [73]. Additionally, compound **86** and dibutyl phthalate (DBP) (**87**) were isolated from the extract broth of marine-derived *B. polymyxa* L_1_-9, which was collected in a mud sample from the intertidal mudflat in the Lianyungang Port of China [74].

**Figure 10:**
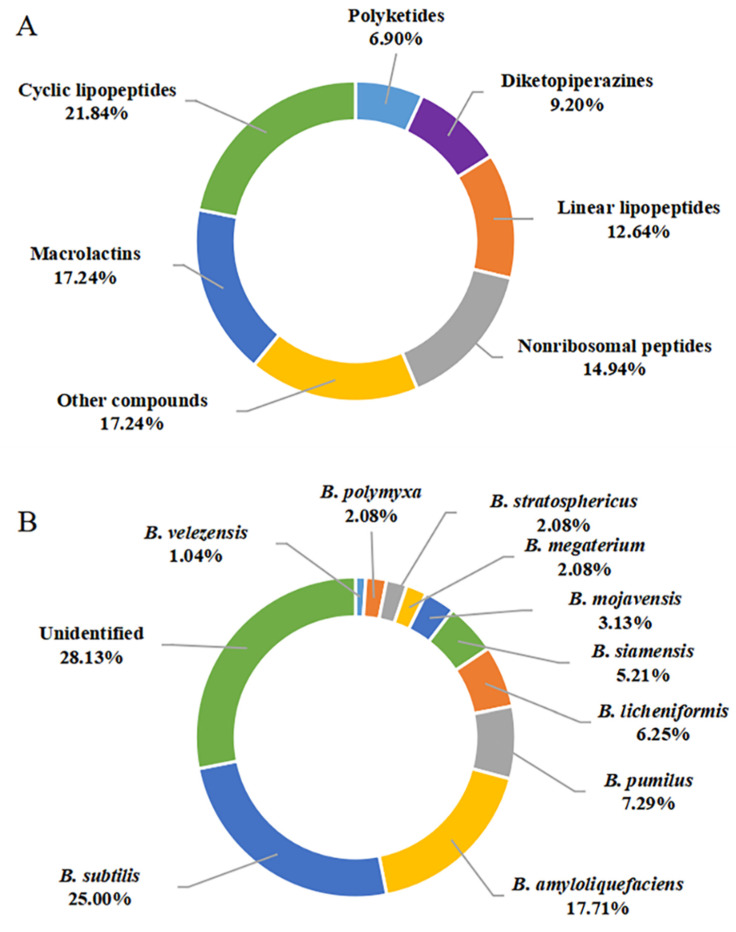

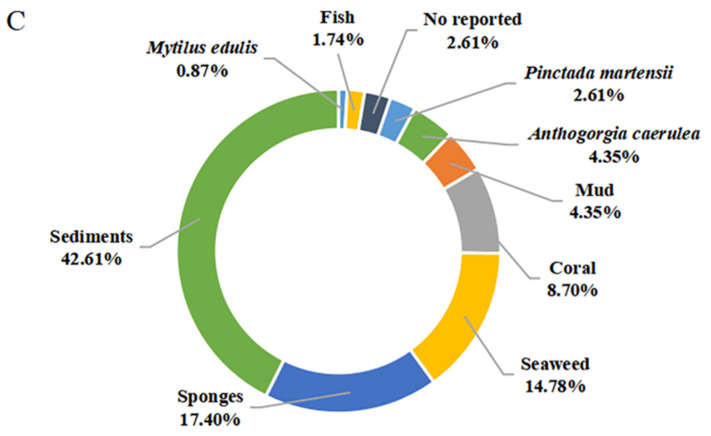
Quantification of this studies on secondary metabolites from marine-derived *Bacillus*: (**A**) Chemical structures categories; (**B**) Producing strains; (**C**) Environment sources.

In this review, a total of 87 secondary metabolites were reported from marine-derived *Bacillus* species from January 2014 to December 2021. Their chemical structures were classified into cyclic lipopeptides (**1**–**19**, among them, **1**–**2** surfactins, **3**–**10** belong to iturins, **11** belongs to plipastatin), diketopiperazines (**20**–**27**), linear lipopeptides (**28**–**38**), nonribosomal peptides (**39**–**51**), polyketides (**52**–**57**), macrolactins (**58**–**72**, among them, **69**–**72** belong to difficidins), and other compounds (**73**–**87**) according to their putative biogenetic sources. As shown in Figure 10A, 21.84% of the compounds reported were CLPs, and these compounds account for an overwhelming majority of all 87 metabolites, followed by macrolactins with 17.24%. Therefore, CLPs are a class of secondary metabolites with structural diversity and pharmacological perspective.

The genus *Bacillus* comprises more than 350 species, some of which are used as antifungal agents, while others are promising producers of green pesticide [14]. As discussed above, a total of 10 identified species, including *B. subtilis*, *B. amyloliquefaciens*, *B. megaterium* CGMCC7086, *B. mojavensis* B0621A, *B. licheniformis*, *B. siamensis*, *B. stratosphericus*, *B. pumilus* RJA1515, *B. polymyxa* L_1_-9, and *B. velezensis* SH-B74 were reported as the producing strains of these described secondary metabolites. Among them, *B. subtilis* and *B. amyloliquefaciens* were the most prolific strains, with 24 (20.00%) and 17 (17.71%) metabolites identified, respectively (Figure 10B). CLPs are ubiquitous in several *Bacillus* strains. However, linear lipopeptides are found predominantly in *B. subtilis* species, while the macrolactins are more preponderant in *B. amyloliquefaciens* species, suggesting the species-specific metabolites.

The secondary metabolites of marine-derived *Bacillus* species could be isolated from marine sediments, marine invertebrates (sponges, molluscs, and corals), and vertebrates (mainly fishes), as well as marine plants (mainly seaweed). Currently, there are 9 reported sources of *Bacillus* secondary metabolites. As shown in Figure 10C, a total of 49 compounds {**1**–**7**,**11**,**15**–**18**,**28**–**37**,**39**–**48**,**58**–**62**,**75**–**85**,**86** (*B. subtilis* AD35)} originated from marine sediments, accounting for 42.61% of the sources of *Bacillus* species. In particular, the producing strains of **58**–**62** originated from deep-sea sediment at a depth of 3000 m, while the other strains of **75**–**83** originated from deep-sea sediment at depths less than 100 m. Moreover, the producing strains of **12**–**14, 86** (*B. polymyxa* L_1_-9), and **87** originated from mud. More precisely, the producing strains of **15**–**16** and **28**–**37** originated from the Republic of Korea’s southern reef. Twenty compounds (**20**–**27**,**39**–**48,50,51**), identified from marine sponges, accounted for 17.40% of the reported environmental sources of *Bacillus* secondary metabolites. It is worth noting that **20** belonging to diketopiperazine were isolated unprecedentedly from a sponge-associated microbe. Moreover, **45**–**49**, **52**–**56** and **63**–**67** were also derived from coral, wherein **63**–**67**, whose producing strains were obtained from the gorgonian coral *A. caerulea*, accounted for 4.35% of the reported environmental sources of *Bacillus* secondary metabolites. The **8**–**10** and **19** that originated from pearl oyster *P. martensii* and *M. edulis*, respectively, accounted for 3.48% of the reported ones. A total of 17 secondary metabolites {**38,45**,**47**,**49**,**52**–**57**,**60**,**62** (*B. amyloliquefaciens* MTCC 10456) and **68**–**72**} were identified from *Bacillus* residing in marine plants, accounted for 14.78% of the reported total amount. Thereinto, the producing strains of **52**–**57** originated from brown seaweed, while the producing strains of **45**,**47**,**49**,**60**,**62** (*B. amyloliquefaciens* MTCC 10456), **38**,**52**–**57** and **68**–**72** were collected from seaweed. In addition to these producing strains, only one *Bacillus* strain (*B. megaterium* CGMCC7086), which produced **45** and **49**, was obtained from the intestines of marine fish and accounted for 1.74% of the reported total amount. Unfortunately, the environmental sources of the producing strain of **47** (*B. subtilis* B38), **73** and **74** (*B. stratosphericus*) were not described. From the above analysis, it can be concluded that marine sediments and sponges are more abundant sources of productive strains of marine-derived *Bacillus*, and which deserved much more attention in subsequent chemical studies.

## 3. Biological Activities

The producing strains, environmental sources, and biological activities of bioactive compounds from marine-derived *Bacillus* are listed. Most compounds possess a range of moderate to potent biological activities, including antimicrobial, antifungal, anticancer and other activities. Detailed descriptions of compounds with promising bioactivities are described as follows.

### 3.1. Antimicrobial Activities

Table 1 lists the potential antimicrobial *Bacillus* secondary metabolites. Compounds **2**–**5** exhibited antibacterial activity against *Vibrio parahaemolyticus* with a minimum inhibitory concentration (MIC) of 50 µg/mL. At the same time, they also displayed significant antibacterial activity against *Escherichia coli*, *Vibrio cholerae*, *Vibrio harveyi*, *Pseudomonas aeruginosa*, *Staphylococcus aureus*, and *Proteus species*, fully illustrating their broad-spectrum bacteriostatic characters [47]. Anti-staphylococcal activity of compounds **15** and **16** was evaluated against clinically methicillin resistant *S. aureus* (MRSA) strains (ATCC25923, XU212, SA1199B, RN4220) with MIC values from 8 to 32 µg/mL by the broth dilution assay, indicating better potential than that of the positive control norfloxacin (MIC, 2–64 µg/mL) [12]. Compounds **17** and **18** exhibited moderate antibacterial activity against Gram-positive bacteria (*S. aureus*, *B. subtilis*)*,* and Gram-negative bacteria (*Salmonella typhi and P. aeruginosa*) with MIC values ranging from 16 to 32 µg/mL, compared to azithromycin (MIC, 2 µg/mL) [50]. Similarly, compounds **28**–**37** were active with MICs of 3–64 µg/mL when tested for the same bacteria as above, which proved they could be good biological probes for antimicrobial agents [35,57,58]. Among them, mixed compounds **28** + **29** were more active than individual ones [35]. Surprisingly, compound **19** displayed inhibitory activity against the clinically relevant strains *S. aureus* and *Enterococcus faecalis* with MIC values of 12 and 8 µM, respectively, compared to the positive control streptomycin (MIC, 2.09 µM and 5.24 µM), demonstrating the potential of marine microorganism as a hopeful source for the development of new antibiotics [51]. Moreover, compound **20** displayed strong antimicrobial activities against *E. coli* and *S. aureus* with MIC values of 16 µg/mL and 22 µg/mL, respectively, compared with ampicillin as positive control. Notably, this was the first report of antibacterial activity of diketopiperazine [53]. Compound **25** exhibited significant antimicrobial activity against fish pathogens, where the MIC was 31.25 mg/mL determined by the broth dilution assay method [55]. Compounds **52** and **53** were reported to display significant antibacterial activity against *V. parahaemolyticus* ATCC^®^ 17802™, *Vibrio vulnificus*, and *Aeromonas hydrophila* [64]. Compounds **54**–**57** exhibited antibacterial activities against human important clinical pathogens *V. vulnificus* and *V. parahaemolyticus* (inhibitory zone diameter greater than 15 mm, 100 mcg on disk) [63]. A new macrolactin **68** showed a moderate effect on *A. hydrophila*, *V. parahemolyticus* ATCC 17802 and *V. vulnificus* with inhibitory zone diameters of 18, 16, and 14 mm at concentration of 100 µg on disk compared with that of the control commercial antibiotics [68]. Difficidin analogues **69**–**72** displayed significant antibacterial activities against multidrug-resistant (MDR) bacteria containing methicillin-resistant *S. aureus,* vancomycin-resistant *E. faecalis*, and other drug-resistant strains, such as *P. aeruginosa* and *Klebsiella pneumonia* with the MICs of 0.002–0.009 µM, compared to positive controls (chloramphenicol and ampicillin with MIC of about 0.017–0.049 µM). Thereinto, a drug-likeness score of **70** was greater than those of other difficidin analogues, demonstrating its potential for pharmaceutical uses against the bottleneck of drug-resistant pathogens [13]. Compounds **73** and **74** exhibited higher activities against *E. faecalis* with MIC values of 0.05–0.8 µg/mL than the positive control (vancomycin and linezolid). In contrast, the antibacterial activities of **73** and **74** against *V. parahaemolyticus*, *Photobacterium damselae*, *Shewanella algae*, *Bacillus amyloliquefaciens ssp-plantarum*, *and Pseudomonas stutzeri* (0.5–8 µg/mL) were lower than those of the positive controls. Both compounds did not show cytotoxicity up to a concentration of 10 µM in the MTT assay [69]. Compounds **75**–**78** displayed antibacterial activity against *P. aeruginosa* PA-01 and *Acinetobacter baumannii* ATCC19606 with MIC values 58–64 µg/mL, while the positive control ofloxacin showed MIC values of 1–16 µg/mL [70]. Compounds **82** and **83** displayed good antimicrobial activities against *P. aeruginosa*, *E. coli*, *Bacillus cereus*, *B. subtilis*, *S. typhi*, and *S. aureus* with MICs values of 0.01–0.05 µM, while azithromycin exhibiting an MIC value of 0.003 µM [71]. Compound **86** displayed broad-spectrum antimicrobial activities against *Salmonella typhimurium*, MRSA, *Listeria monocytogenes*, *A. hydrophila*, *S. aureus*, *Staphylococcus epidermidis*, *E. coli*, and *P. aeruginosa* with MICs as follows: 16 µg/mL, 32 µg/mL, 0.25 µg/mL, 0.5 µg/mL, 8 µg/mL, 4 µg/mL, 4 µg/mL, and 8 µg/mL. Additionally, the minimum bactericidal concentration (MBC) values of **86** observed ranged from 1 to 64 µg/mL, while the lowest MBC value of **86** was 1 µg/mL for *L. monocytogenes* followed by *A. hydrophila* (2 µg/mL), *E. coli* (8 µg/mL), *S. aureus* (16 µg/mL) and *S. epidermidis* (16 µg/mL). On the other hand, the highest MBC values were obtained with MRSA (64 µg/mL), *S. typhimurium* (32 µg/mL) and *P. aeruginosa* (32 µg/mL) [73]. Compounds **86** and **87** exhibited inhibitory zone diameters of 1.3–9.7 mm against *E. coli*, *B. subtilis*, and *S. aureus*, wherein **87** showed higher inhibitory effect than **86**, which proved they could be used as potential candidates for new antibacterial agents [74].

### 3.2. Antifungal Activities

The *Bacillus* secondary metabolites with potential antifungal activities are listed in Table 2. It was the first time reported that a surfactin type CLP **1** displayed an inhibitory effect on appressorium formation of rice blast causal pathogen *Magnaporthe oryzae* at concentrations of 10 and 50 µM, indicating that **1** may be considered as potential green pesticide against *M. oryzae* as prospected [46]. Compound **6** had antagonistic activities against several plant pathogens (*Valsa mali*, *Fusarium oxysporum* f.sp. *cucumerinum* and *Rhizoctonia solani*) when the concentration was over 2 mg/mL by the paper disc-agar diffusion assay [22]. Compounds **8**–**10** displayed moderate and dose-dependent inhibition of growth with tested *F. oxysporum* (inhibitory zone diameter 5.31–7.33 mm) via paper disc-agar diffusion assay, when the concentrations ranged from 0.5 to 2.0 mM. The difference in antifungal activity between **8** and the other two compounds was statistically significant (*p* < 0.05). With respect to activity–structure relationships, a conclusion can be drawn that antifungal activity is closely related to the length of fatty acid chains, as well as C16 and C17 forms of mojavensins [48]. Research revealed that compound **11** has excellent activity in vitro on the suppression of the conidia germination of *Botrytis cinerea* (MIC, 50 µM) [49]. Compounds **17** (half maximal inhibitory concentration, IC_50_ = 1 µg/mL) was an approximately 400-times-stronger inhibitor than compound **18** (IC_50_ = 400 µg/mL) on the motility of zoospores of *Phytophthora capsici* with dose-dependent and time-dependent manners. It is also noteworthy that the zoospores blocked by **17** whereafter cleaved at a higher concentration (IC_50_ = 50 µg/mL). It manifested that the methyl group at C-12 of the fatty acid in **17** is vital for activity. Therefore, compound **17** can be used to develop fungicides targeting *P. capsici* [50]. Moreover, compounds **17** and **18** possessed promising inhibitory activities against *R. solani, Colletotrichum acutatum*, *B. cinerea* with MIC values ranging from 4 to 8 µg/mL, which were comparable to that of amphotericin B (MIC, 1 µg/mL) [50]. Similarly, compounds **28**–**37** were active with MICs of 1–32 µg/mL when testing the same pathogenic fungi as above [35,57,58]. Among them, mixed compounds **28** + **29** were more active than individual ones. Additionally, the four novel non-cytotoxic lipopeptides **34**–**37** from marine-derived *B. subtilis* highlighted the research for novel safe antifungal agents [35,58]. Compounds **32**, **34**–**37** could inhibit mycelial growth, conidiogenesis, conidia germination, morphological alterations in the germinated conidia, and wheat blast disease. Additionally, the MICs of the compounds **32**, **34**–**37** were as follows: 1.5 µg/disk (**32**), 2.5 µg/disk (**36**), 2.5 µg/disk (**37**), 10.0 µg/disk (**34**), and 10.0 µg/disk (**35**), revealing **32** showed the highest mycelial growth inhibition of *M. oryzae Triticum* (MoT) among the tested compounds [75]. Compounds **45**, **47** and **49** displayed antifungal activity against filamentous fungi and yeasts [45]. Additionally, compound **47** also exhibited antifungal activity against *Candida albicans* with an MIC value of 12.5 µg/mL [61]. Compounds **38**, **60** and **62** showed significant antifungal activity against *Malassezia* spp. (*Malassezia furfur* ATCC 44344, *Malassezia furfur* ATCC 44344, and *Malassezia globosa* ATCC MYA 4612) with MIC values ranging from 38 to 330 µg/mL, while the positive control (ketoconazole) had MIC values ranging from 0.03 to 0.08 µg/mL. Therefore, these compounds reported against *Malassezia* spp. could be beneficial for application in the field of cosmetics and dermatology [44]. Macrolactins A-E (**63**–**67**) displayed significant antagonistic activities against *Sporisorium scitamineum* with EC_50_ values (for 50% of maximal effect) of 0.31–67.99 µg/mL, wherein **65** displayed the highest potency against *S. scitamineum* compared with azoxystrobin (EC_50_, 0.26–36.66 µg/mL). The study of structure and activity relationship proved that a hydroxyl group at C-20 and a glycosyl group at C-7 play an important role in **65**. Additionally, **65** inhibited the proliferation and mycelial growth of *S. scitamineum* spores with EC_50_ values of 0.52, and 3.25 μg/mL, respectively [68]. Compounds **82** and **83** exhibited good inhibitory effect on the plant pathogenic fungi *B. cenerea*, *R. solani*, *C. acutatum*, and *Aspergillus niger*, with MICs ranging from 0.03 to 0.05 µM compared to positive control amphotericin B (MIC, 0.003 µM). Moreover, **82** and **83** were reported to possess promising antifungal activity against human pathogen *C. albicans* with MICs ranging from of 0.03 to 0.05 µM, compared with positive control amphotericin B (MIC, 0.003 µM) [71]. Compounds **86** and **87** showed inhibitory zone diameter of 5.3–11.0 mm against *Sclerotina sclerotiorum*, *Bipolaris sorokiniana*, *R. solani*, *Alternaria solani*, and *Bipolaris maydis* [74].

### 3.3. Cytotoxic Activities

The potential cytotoxic *Bacillus* secondary metabolites are listed in Table 3. Compound **12** displayed moderate cytotoxicity against the cancer cell lines including SK-HEP-1 (liver cancer), HCT116 (colorectal cancer), MDA-MB-231 (breast cancer), A549 (lung cancer), SNU638 (stomach cancer) and antimetastatic activity against breast cancer cells (MDA-MB-231) with IC_50_ ranging from of 28 to 39 µM compared to positive control Etoposide (IC_50_, 0.42–6.21 µM). By contrast, compounds **13** and **14** did not exhibit antiproliferative and antimetastatic activities against human cancer cells, which indicated 2-N-OH, 16-N-OH, 37-OH (carboxylic acid) in **12** play an important role**.** More rarely, **12** was inactive towards normal epithelial cells (IC_50_ > 50µM) [25]. Compounds **28**–**30** were cytotoxic towards breast cancer (MDA-MB-231), colon cancer (HCT-15), prostate cancer (PC-3), lung cancer (NCI-H23), stomach cancer (NUGC-3), and renal cancer (ACHN), with GI_50_ values of 4.6 to 23.2 µg/mL compared with the positive control Adriamycin (GI_50_, 0.33 to 0.91 µg/mL). It is noteworthy that mixed compounds **28** + **29** demonstrated better activity than individuals, especially for lung cancer (NCI-H23) with the GI_50_ value of 4.6 µg/mL [35]. Compounds **45**–**48** were observed with moderate cytotoxic activities towards human cancer cell lines MCF7 (breast cancer) and HepG2 (liver cancer) with IC_50_ values ranging from 8.2 ± 0.2 to 2.9 ± 0.1 µM, whereas compounds **39**–**44** had no cytotoxic effects, suggesting that a C16-fatty acid of bacillomycin D analogues may be the key moiety with best cytotoxicities [60]. The MTS assay illustrated that compound **58** can promote cell proliferation at the concentration of 10 µM and **58**–**62** did not display obvious cytotoxic effect in the murine macrophage cell line RAW 264.7 at the employed concentrations (10–40 µM) [66]. Compound **84** possessed the inhibitory effects on human melanoma cell line MNT-1 and melanin synthesis in mouse melanoma cell line B16F10 [72]. Compound **85** displayed enzyme inhibitory activity against melanogenic enzymes, which could modulate melanogenesis by down-regulating melanogenic enzymes expression with minimal cytotoxicity in murine melanoma cells, such as MNT-1, B16F10 and human melanoma cell [72].

### 3.4. Other Activities

In the previous study, sponge epiphytic *Bacillus* was found to have an obvious inhibitory effect on the attachment of many kinds of diatoms. Compounds **21**–**27** could inhibit the attachment of diatoms to a certain extent in order to help the host sponges realize the chemical defense to *Nitzschia closterium*, so they can be used as marine natural products antifouling agent with high possibility and selectivity [54]. Compounds **50** and **51** exhibited siderophore activity at concentrations ranging from 1.28 mM to 1.25 µM, which were the first bacterial siderophores containing benzoic acid and nicotinic acid moieties [24]. Owing to the existence of the epoxy ring, compound **58** exhibited a significant inhibitory effect on the expression of various cytokines inducible and nitric oxide, compared with previously known macrolactins (**59**–**62**). Additionally, **58** reduced the mRNA expression level of IL-1*β* with concentration-dependent manner [66]. Of note, in addition to cytotoxicity in melanoma cells, the anti-pigmentary effect of **85** was demonstrated in an artificial human skin model with comparable or superior effects to those of arbutin, bisabolol and kojic acid, indicating it could be potential as hypopigmenting agent [72].

**Figure 11:**
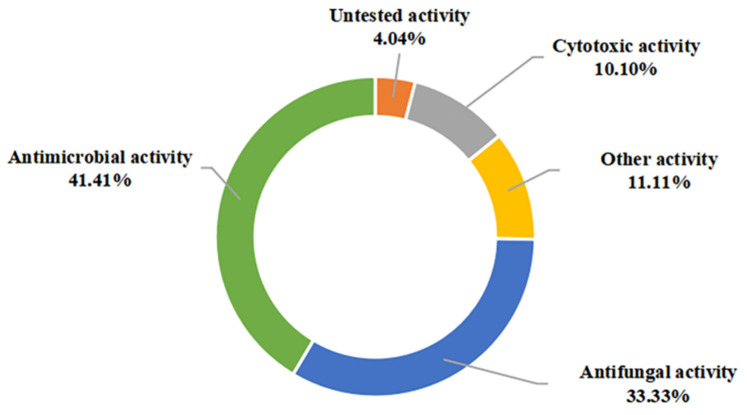
Bioactivity categories of the secondary metabolites from marine-derived *Bacillus*.

In this view, most of the presented compounds display significant biological activities such as antimicrobial, antifungal, and anticancer. These compounds are also effective to a certain extent in inhibiting the attachment of diatoms, the expression of various cytokines inducible and nitric oxide exhibited siderophore activity, as well as enzyme inhibitory activity. Among them, antimicrobial (41.41%), antifungal (33.33%), and anticancer (10.10%) activities were dominant in evaluating the pharmacological potential of these metabolites (Figure 11). Notably, a high proportion (54.84%) of the 49 marine sediment-derived metabolites exhibited moderate to potent bioactivity. Based on this, these impressive bioactivities indicate their potential to be new antibiotic candidates, biological control agents, and hypopigmenting agents.

## 4. Conclusions

This mini-review summarizes a total of 87 secondary metabolites produced by marine-derived *Bacillus* species reported in recent years. The described compounds were organized on the basis of their structural diversity, and biological activities. CLPs are ubiquitous in several *Bacillus* strains, meanwhile linear lipopeptides and macrolactins are found predominantly in *B. subtilis* and *B. amyloliquefaciens* species as species-specific metabolites. In addition, marine sediments and sponges are more abundant sources of productive strains of marine-derived *Bacillus*, and most marine sediment-derived metabolites exhibited moderate to potent bioactivity, which deserves much more attention in subsequent chemical studies. Therefore, marine-derived *Bacillus* species are a potential promising source for the discovery of new metabolites.

## Figures and Tables

**Table 1:** The antimicrobial activities of the secondary metabolites from marine-derived *Bacillus*.

NO.	Classification	Producing Strain	Environmental Source	Antimicrobial Activities ^a^	Ref.
**2**	Cyclic lipopeptide (surfactin)	*B. licheniformis* MB01	sediments (the Bohai Sea, China)	*V. parahaemolyticus* (>3 cm), *E. coli* (>3 cm), *V. cholerae* (>3 cm), *V. harveyi* (>3 cm), *P. aeruginosa* (2–3 cm), *S. aureus* (2–3 cm), *Proteus species* (1–2 cm) (inhibition zone diameter)	[47]
**3**	Cyclic lipopeptide (surfactin)	*B. licheniformis* MB01	sediments (the Bohai Sea, China)	*V. parahaemolyticus* (>3 cm), *E. coli* (>3 cm), *V. cholerae* (>3 cm), *V. harveyi* (>3 cm), *P. aeruginosa* (2–3 cm), *S. aureus* (2–3 cm), *Proteus species* (1–2 cm) (inhibition zone diameter)	[47]
**4**	Cyclic lipopeptide (surfactin)	*B. licheniformis* MB01	sediments (the Bohai Sea, China)	*V. parahaemolyticus* (>3 cm), *E. coli* (>3 cm), *V. cholerae* (>3 cm), *V. harveyi* (>3 cm), *P. aeruginosa* (2–3 cm), *S. aureus* (2–3 cm), *Proteus species* (1–2 cm) (inhibition zone diameter)	[47]
**5**	Cyclic lipopeptide (surfactin)	*B. licheniformis* MB01	sediments (the Bohai Sea, China)	*V. parahaemolyticus* (>3 cm), *E. coli* (>3 cm), *V. cholerae* (>3 cm), *V. harveyi* (>3 cm), *P. aeruginosa* (2–3 cm), *S. aureus* (2–3 cm), *Proteus species* (1–2 cm) (inhibition zone diameter)	[47]
**15**	Cyclic lipopeptide	*B. subtilis* 109GGC020	sediments (the Gageocho reef, Korea)	MRSA ATCC25923 (8 μg/mL), MRSA XU2120 (16 μg/mL), MRSA SA1199B (8 μg/mL), MRSA RN4220 (32 μg/mL) (MIC)	[12]
**16**	Cyclic lipopeptide	*B. subtilis* 109GGC020	sediments (the Gageocho reef, Korea)	MRSA ATCC25923 (16 μg/mL), MRSA XU2120 (16 μg/mL), MRSA SA1199B (32 μg/mL) (MIC)	[12]
**17**	Cyclic lipopeptide	*B. subtilis* 109GGC020	sediments (the Gageocho reef, Korea)	*S. aureus* (16 μg/mL), *B. subtilis* (16 μg/mL)*, S. typhi* (16 μg/mL), *P. aeruginosa* (16 μg/mL) (MIC)	[50]
**18**	Cyclic lipopeptide	*B. subtilis* 109GGC020	sediments (the Gageocho reef, Korea)	*S. aureus* (16 μg/mL), *B. subtilis* (32 μg/mL), *S. typhi* (32 μg/mL), *P. aeruginosa* (16 μg/mL) (MIC)	[50]
**19**	Cyclic lipopeptide	*Bacillus* sp. BC028	the blue mussel *M. edulis* (the western shore of Baltic Sea, Germany)	*S. aureus* NCTC 8325 (12 μM), *E. faecalis* JH212 (8 μM) (MIC)	[51]
**20**	Cyclic lipopeptide	*Bacillus* sp. SPB7	the sponge *S. officinali* (the Palk Bay of Bengal, Mandapam coast, Tamil Nadu, India)	*E. coli* (16 μg/mL), *S. aureus* (22 μg/mL) (MIC)	[53]
**25**	Pyrrol	*Bacillus* sp.	sponges (Agatti island,	fish pathogens (GI_50_, 31.25 µg/mL)	[55]
			Lakshadweep archipelago, India)		
**28**	Linear lipopeptide	*B. subtilis* 109GGC020	sediments (the Gageocho reef, Korea)	*S. typhi* (16 μg/mL), *S. aureus* (16 μg/mL), *P. aeruginosa* (16 μg/mL), *B. subtilis* (16 μg/mL) (MIC)	[35]
**29**	Linear lipopeptide	*B. subtilis* 109GGC020	sediments (the Gageocho reef, Korea)	*S. typhi* (32 μg/mL), *S. aureus* (16 μg/mL), *P. aeruginosa* (16 μg/mL), *B. subtilis* (32 μg/mL) (MIC)	[35]
**30**	Linear lipopeptide	*B. subtilis* 109GGC020	sediments (the Gageocho reef, Korea)	*S. typhi* (32 μg/mL), *S. aureus* (64 μg/mL), P. aeruginosa (64 μg/mL), *B. subtilis* (32 μg/mL) (MIC)	[35]
**31**	Linear lipopeptide	*B. subtilis* 109GGC020	sediments (the Gageocho reef, Korea)	*S. aureus* (0.03 µM), *B. subtilis* (0.03 µM), *S. typhi* (0.06 µM), *P. aeruginosa* (0.06 µM) (MIC)	[57]
**32**	Linear lipopeptide	*B. subtilis* 109GGC020	sediments (the Gageocho reef, Korea)	*S. aureus* (0.04 µM), *B. subtilis* (0.02 µM), *S. typhi* (0.02 µM), *P. aeruginosa* (0.04 µM) (MIC)	[57]
**33**	Linear lipopeptide	*B. subtilis* 109GGC020	sediments (the Gageocho reef, Korea)	*S. aureus* (0.04 µM), *B. subtilis* (0.04 µM), *S. typhi* (0.02 µM), *P. aeruginosa* (0.02 µM) (MIC)	[57]
**34**	Linear lipopeptide	*B. subtilis* 109GGC020	sediments (the Gageocho reef, Korea)	*S. aureus* (0.05 µM), *B. subtilis* (0.05 µM), *S. typhi* (0.05 µM), *P. aeruginosa* (0.09 µM) (MIC)	[58]
**35**	Linear lipopeptide	*B. subtilis* 109GGC020	sediments (the Gageocho reef, Korea)	*S. aureus* (0.05 µM), *B. subtilis* (0.05 µM), *S. typhi* (0.08 µM), *P. aeruginosa* (0.08 µM) (MIC)	[58]
**36**	Linear lipopeptide	*B. subtilis* 109GGC020	sediments (the Gageocho reef, Korea)	*S. aureus* (0.08 µM), *B. subtilis* (0.09 µM), *S. typhi* (0.09 µM), *P. aeruginosa* (0.09 µM) (MIC)	[58]
**37**	Linear lipopeptide	*B. subtilis* 109GGC020	sediments (the Gageocho reef, Korea)	*S. aureus* (0.08 µM), *B. subtilis* (0.05 µM), *S. typhi* (0.09 µM), *P. aeruginosa* (0.08 µM) (MIC)	[58]
**52**	Polyketide	*B. subtilis*	the brown seaweed *S. myriocystum* (Mannar Bay on the southeast coast of India)	*V. parahemolyticus* (24.33 ± 0.58 mm, 10 μg on disk), *V. vulnificus* (22.66 ± 0.58 mm)*, A. hydrophila* (26.00 ± 1.00 mm) (inhibition zone diameter)	[64]
		MTCC 10407			
**53**	Polyketide	*B. subtilis*	the brown seaweed *S. myriocystum* (Mannar Bay on the southeast coast of India)	*V. parahemolyticus* (11.00 ± 1.00 mm), *V. vulnificus* (22.66 ± 0.58 mm), *A. hydrophila* (17.66 ± 0.58 mm) (inhibition zone diameter)	[64]
		MTCC 10407			
**54**	Polyketide	*B. amyloliquefaciens*	the brown seaweed *P. gymnospora* (Mannar Bay, Peninsular India)	*V. vulnificus* MTCC 1145 (16.33 ± 0.58 mm, 10 mcg on disk), *V. parahaemolyticus* ATCC^®^ 17802™ (15.30 ± 1.15 mm), *A. hydrophila* MTCC 646 (12.67 ± 1.15 mm), *V. harveyi* MTCC 3438 (15.33 ± 0.58 mm), *V. anguillarum* (13.33 ± 1.15 mm), *V. parahaemolyticus* MTCC 451 (14.00 ± 1.00 mm) (inhibition zone diameter)	[63]
**55**	Polyketide	*B. amyloliquefaciens*	the brown seaweed *P. gymnospora* (Mannar Bay, Peninsular India)	*V. vulnificus* MTCC 1145 (14.67 ± 1.15 mm), *V. parahaemolyticus* ATCC^®^ 17802™ (14.00 ± 1.00 mm), *A. hydrophila* MTCC 646 (14.67 ± 1.15 mm), *V. harveyi* MTCC 3438 (13.00 ± 1.00 mm), *V. anguillarum* (12.67 ± 1.54 mm), *V. parahaemolyticus* MTCC 451 (13.33 ± 1.15 mm) (inhibition zone diameter)	[63]
**56**	Polyketide	*B. amyloliquefaciens*	the brown seaweed *P. gymnospora* (Mannar Bay, Peninsular India)	*V. vulnificus* MTCC 1145 (17.33 ± 1.15 mm), *V. parahaemolyticus* ATCC^®^ 17802™ (16.00 ± 1.00 mm), *A. hydrophila* MTCC 646 (13.33 ± 1.15 mm), *V. harveyi* MTCC 3438 (12.67 ± 1.15 mm), *V. anguillarum* (10.66 ± 1.15 mm), *V. parahaemolyticus* MTCC 451 (15.33 ± 0.58 mm) (inhibition zone diameter)	[63]
**57**	Polyketide	*B. amyloliquefaciens*	the brown seaweed *P. gymnospora* (Mannar Bay, Peninsular India)	*V. vulnificus* MTCC 1145 (13.67 ± 1.52 mm), *V. parahaemolyticus* ATCC^®^ 17802™ (12.66 ± 1.15 mm), *A. hydrophila* MTCC 646 (11.33 ± 1.15 mm), *V. harveyi* MTCC 3438 (15.33 ± 0.58 mm), *V. parahaemolyticus* MTCC 451 (12.67 ± 1.54 mm) (inhibition zone diameter)	[63]
**68**	Macrolactin	*B. subtilis* MTCC10403	the seaweed *Anthophycus longifolius* (Mannar Bay, Peninsular India)	*A. hydrophilla* (18 mm, 100 μg on disk*)*, *V. parahemolyticus* ATCC 17802 (16 mm), *V. vulnificus* (14 mm) (inhibition zone diameter)	[68]
**69**	Macrolactin (difficidin)	*B. amyloliquefaciens* MTCC12713	an intertidal macroalga *Kappaphycus alverezii* (Mannar Bay, Peninsular India)	MRSA ATCC33592 (0.005 µM), VRE*fs* ATCC51299 (0.009 µM), *P. aeruginosa* ATCC27853 (0.006 µM), *K. pneumonia* ATCC13883 (0.009 µM), *E. tarda* MTCC2400 (0.005 µM), *E. coli* MTCC443 (0.009 µM), *S. pyogenes* MTCC1924 (0.009 µM), *V. parahaemolyticus* MTCC451 (0.006 µM) (MIC)	[13]
**70**	Macrolactin (difficidin)	*B. amyloliquefaciens* MTCC12713	an intertidal macroalga *Kappaphycus alverezii* (Mannar Bay, Peninsular India)	MRSA ATCC33592 (0.002 µM), VRE*fs* ATCC51299 (0.002 µM), *P. aeruginosa* ATCC27853 (0.004 µM), *K. pneumonia* ATCC13883 (0.002 µM), *E. tarda* MTCC2400 (0.004 µM), *E. coli* MTCC443 (0.004 µM), *S. pyogenes* MTCC1924 (0.007 µM), *V. parahaemolyticus* MTCC451 (0.004 µM) (MIC)	[13]
**71**	Macrolactin (difficidin)	*B. amyloliquefaciens* MTCC12713	an intertidal macroalga *Kappaphycus alverezii* (Mannar Bay, Peninsular India)	MRSA ATCC33592 (0.002 µM), VRE*fs* ATCC51299 (0.004 µM), *P. aeruginosa* ATCC27853 (0.002 µM), *K. pneumonia* ATCC13883 (0.002 µM), *E. tarda* MTCC2400 (0.004 µM), *E. coli* MTCC443 (0.004 µM), *S. pyogenes* MTCC1924 (0.005 µM), *V. parahaemolyticus* MTCC451 (0.002 µM) (MIC)	[13]
**72**	Macrolactin (difficidin)	*B. amyloliquefaciens* MTCC12713	an intertidal macroalga *Kappaphycus alverezii* (Mannar Bay, Peninsular India)	MRSA ATCC33592 (0.002 µM), VRE*fs* ATCC51299 (0.004 µM), *P. aeruginosa* ATCC27853 (0.002 µM), *K. pneumonia* ATCC13883 (0.002 µM), *E. tarda* MTCC2400 (0.004 µM), *E. coli* MTCC443 (0.004 µM), *S. pyogenes* MTCC1924 (0.004 µM), *V. parahaemolyticus* MTCC451 (0.002 µM) (MIC)	[13]
**73**	Thiopeptide	*B. stratosphericus*	no description	*S. aureus* KCTC 1927 (0.8 µg/mL), *K. rhizophila* KCTC 1915 (0.2 µg/mL), *B. subtilis* KCTC 1021 (0.8 µg/mL), *E. coli* KCTC 2441 (26 µg/mL), *K. pneumoniae* KCTC 2690 (26 µg/mL), *S. typhimurium* KCTC 2515 (26 µg/mL) (MIC)	[69]
**74**	Thiopeptide	*B. stratosphericus*	no description	*S. aureus* KCTC 1927 (0.1 µg/mL), *K. rhizophila* KCTC 1915 (0.05 µg/mL), *B. subtilis* KCTC 1021 (0.5 µg/mL), *E. coli* KCTC 2441 (26 µg/mL), *K. pneumoniae* KCTC 2690 (26 µg/mL), *S. typhimurium* KCTC 2515 (26 µg/mL) (MIC)	[69]
**75**	Long-chain amide	*B. pumilus* RJA1515	sediments (at a depth of 84 m, Bamfield, British Columbia)	*P. aeruginosa* PA-01 (64 µg/mL), *A. baumannii* ATCC19606 (58 µg/mL) (MIC)	[70]
**76**	Long-chain amide	*B. pumilus* RJA1515	sediments (at a depth of 84 m, Bamfield, British Columbia)	*P. aeruginosa* PA-01 (64 µg/mL), *A. baumannii* ATCC19606 (64 µg/mL) (MIC)	[70]
**77**	Long-chain amide	*B. pumilus* RJA1515	sediments (at a depth of 84 m, Bamfield, British Columbia)	*P. aeruginosa* PA-01 (64 µg/mL), *A. baumannii* ATCC19606 (64 µg/mL) (MIC)	[70]
**78**	Long-chain amide	*B. pumilus* RJA1515	sediments (at a depth of 84 m, Bamfield, British Columbia)	*P. aeruginosa* PA-01 (64 µg/mL), *A. baumannii* ATCC19606 (58 µg/mL) (MIC)	[70]
**82**	Glycolipid	*B. licheniformis* 09IDYM23	sediments (at a depth 20 m, Ieodo, Korea)	*P. aeruginosa* (0.01 µM), *E. coli* (0.01 µM), *B. cereus* (0.01 µM), *B. subtilis* (0.03 µM), *S. typhi* (0.01 µM), *S. aureus* (0.03 µM) (MIC)	[71]
**83**	Glycolipid	*B. licheniformis* 09IDYM23	sediments (at a depth 20 m, Ieodo, Korea)	*P. aeruginosa* (0.03 µM), *E. coli* (0.03 µM), *B. cereus* (0.03 µM), *B.subtilis* (0.05 µM), *S. typhi* (0.05 µM), *S. aureus* (0.03 µM) (MIC)	[71]
**86**	Other compounds	*B. subtilis* AD35	marine water and sediment (Alexandria sea shore, Egypt)	*S. typhimurium* (16 µg/mL), MRSA (32 µg/mL), *L. monocytogenes* (0.25 µg/mL), *A. hydrophila* (0.5 µg/mL), *S. aureus* (8 µg/mL), *S. epidermidis* (4 µg/mL), *E. coli* (4 µg/mL), *P. aeruginosa* (8 µg/mL) (GI_50_)	[73]
		*B. polymyxa* L_1_-9	the mud of the intertidal mudflat (Lianyungang Port, China)	*E. coli* (5.2 ± 0.20 mm), *B. subtilis* (4.3 ± 0.11 mm), *S. aureus* (1.3 ± 0.02 mm) (inhibition zone diameter)	[74]
**87**	Other compounds	*B. polymyxa* L_1_-9	the mud of the intertidal mudflat (Lianyungang Port, China)	*E. coli* (9.7 ± 0.20 mm), *B. subtilis* (6.6 ± 0.16 mm), *S. aureus* (2.3 ± 0.12 mm) (inhibition zone diameter)	[74]

^a^ Only the most competitive values are listed in this table due to limited space.

**Table 2:** The antifungal activities of the secondary metabolites from marine-derived *Bacillus*.

NO.	Classification	Producing Strain	Environmental Source	Antifungal Activities ^a^	Ref.
**1**	Cyclic lipopeptide (surfactin)	*B. velezensis* SH-B74	sediments (CCTCC)	*M. oryzae* (concentration, 10–50 µM)	[46]
**6**	Cyclic lipopeptide (iturin)	*B. amyloliquefaciens* SH-B74	sediments (the South China Sea, China)	*V. mali*, *F. oxysporum* f.sp. *cucumerinum*, *R. solani* (concentration, >2 mg/mL)	[22]
**8**	Cyclic lipopeptide (iturin)	*B. mojavensis* B0621A	the pearl oyster *P. martensii* (the South China Sea, China)	*F. oxysporum* f.sp. *cucumerinum* (1.0 mM, 5.63 ± 0.03 mm; 2.0 mM, 6.41 ± 0.56 mm), *F. oxysporum* f.sp. *vasinfectum* (2.0 mM, 5.94 ± 0.25 mm), *F. oxysporum* f.sp. *vasinfectum* SF2 (2.0 mM, 6.21 ± 0.36 mm) (inhibition zone diameter)	[48]
**9**	Cyclic lipopeptide (iturin)	*B. mojavensis* B0621A	the pearl oyster *P. martensii* (the South China Sea, China)	*F. oxysporum* f.sp. *cucumerinum* (0.5 Mm, 6.45 ± 0.13 mm; 1.0 mM, 6.52 ± 0.30 mm; 2.0 mM, 7.33 ± 0.24 mm), *F. oxysporum* f.sp. *vasinfectum* (0.5 mM, 6.46 ± 0.30 mm; 1.0 mM, 7.07 ± 0.10 mm; 2.0 mM, 7.33 ± 0.07 mm), *F. oxysporum* f.sp. *vasinfectum* SF2 (0.5 mM, 5.62 ± 0.40 mm; 1.0 mM, 6.63 ± 0.43 mm; 2.0 mM, 7.19 ± 0.34 mm), *F. oxysporum* f.sp. *Cucumis melo* L. (2.0 mM, 6.32 ± 0.37 mm) (inhibition zone diameter)	[48]
**10**	Cyclic lipopeptide (iturin)	*B. mojavensis* B0621A	the pearl oyster *P. martensii* (the South China Sea, China)	*F. oxysporum* f.sp. *cucumerinum* (0.5 mM, 5.43 ± 0.23 mm; 1.0 mM, 6.61 ± 0.31 mm; 2.0 mM, 8.02 ± 0.04 mm), *F. oxysporum* f.sp. *vasinfectum* (0.5 mM, 5.31 ± 0.17 mm; 1.0 mM, 6.12 ± 0.43 mm; 2.0 mM, 7.26 ± 0.45 mm), *F. oxysporum* f.sp. *vasinfectum* SF2 (0.5 mM, 5.45 ± 0.24 mm; 1.0 mM, 6.31 ± 0.20 mm; 2.0 mM, 6.89 ± 0.26 mm) (inhibition zone diameter)	[48]
**11**	Cyclic lipopeptide (plipastatin)	*B. amyloliquefaciens* SH-B74	sediments (CCTCC)	*B. cinerea* (MIC, 50 µM)	[49]
**17**	Cyclic lipopeptide	*B. subtilis* 109GGC020	sediments (the Gageocho reef, Korea)	*P. capsici* (IC_50_, 1 μg/mL), *R. solani* (MIC, 4 µg/mL), *C. acutatum* (MIC, 8 µg/mL), *B. cinerea* (MIC, 4 µg/mL)	[50]
**18**	Cyclic lipopeptide	*B. subtilis* 109GGC020	sediments (the Gageocho reef, Korea)	*P. capsici* (IC_50_, 400 μg/mL), *R. solani* (MIC, 8 µg/mL), *C. acutatum* (MIC, 8 µg/mL), *B. cinerea* (MIC, 8 µg/mL)	[50]
**28**	Linear lipopeptide	*B. subtilis* 109GGC020	sediments (the Gageocho reef, Korea)	*C. acutatum* (8 μg/mL), *B. cinerea* (4 μg/mL), *R. solani* (4 μg/mL) (MIC)	[35]
**29**	Linear lipopeptide	*B. subtilis* 109GGC020	sediments (the Gageocho reef, Korea)	*C. acutatum* (8 μg/mL), *B. cinerea* (8 μg/mL), *R. solani* (8 μg/mL) (MIC)	[35]
**30**	Linear lipopeptide	*B. subtilis* 109GGC020	sediments (the Gageocho reef, Korea)	C. acutatum (16 μg/mL), B. cinerea (32 μg/mL), R. solani (32 μg/mL) (MIC)	[35]
**31**	Linear lipopeptide	*B. subtilis* 109GGC020	sediments (the Gageocho reef, Korea)	B. cinerea (0.03 µM), C. acutatum (0.03 µM), R. solani (0.06 µM) (MIC)	[57]
**32**	Linear lipopeptide	*B. subtilis* 109GGC020	sediments (the Gageocho reef, Korea)	B. cinerea (0.01 µM), C. acutatum (0.01 µM), R. solani (0.02 µM) (MIC)	[57]
				MoT (MIC, 1.5 µg/disk)	[75]
**33**	Linear lipopeptide	*B. subtilis* 109GGC020	sediments (the Gageocho reef, Korea)	B. cinerea (0.01 µM), C. acutatum (0.02 µM), R. solani (0.02 µM) (MIC)	[57]
**34**	Linear lipopeptide	*B. subtilis* 109GGC020	sediments (the Gageocho reef, Korea)	R. solani (0.02 µM), B. cinerea (0.06 µM), C. acutatum (0.04 µM) (MIC)	[58]
				MoT (MIC, 10.0 µg/disk)	[75]
**35**	Linear lipopeptide	*B. subtilis* 109GGC020	sediments (the Gageocho reef, Korea)	R. solani (0.04 µM), B. cinerea (0.06 µM), C. acutatum (0.04 µM) (MIC)	[58]
				MoT (MIC, 10.0 µg/disk)	[75]
**36**	Linear lipopeptide	*B. subtilis* 109GGC020	sediments (the Gageocho reef, Korea)	R. solani (0.08 µM), B. cinerea (0.08 µM), C. acutatum (0.06 µM) (MIC)	[58]
				MoT (MIC, 2.5 µg/disk)	[75]
**37**	Linear lipopeptide	*B. subtilis* 109GGC020	sediments (the Gageocho reef, Korea)	R. solani (0.08 µM), B. cinerea (0.04 µM), C. acutatum (0.06 µM) (MIC)	[58]
				MoT (MIC, 2.5 µg/disk)	[75]
**38**	Linear lipopeptide	*B. amyloliquefaciens* MTCC 10456	seaweed (MTCC, Chandigarh, India)	M. furfur ATCC 44344 (50–100 µg/mL), M. furfur ATCC 12078 (50–110 µg/mL), M. globosa ATCC MYA 4612 (30–100 µg/mL) (MIC)	[44]
**47**	Nonribosomal peptide	*B. subtilis* B38	not reported	C. albicans ATCC 10231 (MIC, 12.5 µg/mL)	[61]
**60**	Macrolactin	*B. amyloliquefaciens* MTCC 10456	seaweed (MTCC, Chandigarh, India)	M. furfur ATCC 44344 (156–313 µg/mL), M. furfur ATCC 12078 (165–330 µg/mL), M. globosa ATCC MYA 4612 (138–275 µg/mL) (MIC)	[44]
**62**	Macrolactin	*B. amyloliquefaciens* MTCC 10456	seaweed (MTCC, Chandigarh, India)	M. furfur ATCC 44344 (156–313 µg/mL), M. furfur ATCC 12078 (165–330 µg/mL), M. globosa ATCC MYA 4612 (138–275 µg/mL) (MIC)	[44]
**63**	Macrolactin	*B. siamensis*	the *Anthogorgia caerulea* (Beihai city, Guangxi, China)	*S. scitamineum* (EC_50_, 67.99 μg/mL)	[68]
**64**	Macrolactin	*B. siamensis*	the *Anthogorgia caerulea* (Beihai city, Guangxi, China)	*S. scitamineum* (EC_50_, 12.51 μg/mL)	[68]
**65**	Macrolactin	*B. siamensis*	the *Anthogorgia caerulea* (Beihai city, Guangxi, China)	*S. scitamineum* (EC_50_, 3.25 μg/mL)	[68]
**66**	Macrolactin	*B. siamensis*	the *Anthogorgia caerulea* (Beihai city, Guangxi, China)	*S. scitamineum* (EC_50_, 15.05 μg/mL)	[68]
**67**	Macrolactin	*B. siamensis*	the *Anthogorgia caerulea* (Beihai city, Guangxi, China)	*S. scitamineum* (EC_50_, 34.28 μg/mL)	[68]
**82**	Glycolipid	*B. licheniformis* 09IDYM23	sediments (at a depth 20 m, Ieodo, Korea)	*B. cinerea* (0.03 µM), *R. solani* (0.03 µM), *C. acutatum* (0.03 µM), *A. niger* (0.05 µM) (IC_50_), *C. albicans* (0.05µM) (MIC)	[71]
**83**	Glycolipid	*B. licheniformis* 09IDYM23	sediments (at a depth 20 m, Ieodo, Korea)	*B. cinerea* (0.05 µM), *R. solani* (0.03 µM), *C. acutatum* (0.03 µM), *A.niger* (0.05 µM) (IC_50_), *C. albicans* (0.03µM) (MIC)	[71]
**86**	Other compounds	*B. polymyxa* L_1_-9	the mud of the intertidal mudflat (Lianyungang Port, China)	*S. sclerotiorum* (9.0 ± 0.3 mm), *B. sorokiniana* (8.7 ± 0.10 mm), *R. solani* (7.2 ± 0.11 mm), *A. solani* (6.0 ± 0.13 mm), *B. maydis* (5.3 ± 0.06 mm) (inhibition zone diameter)	[74]
**87**	Other compounds	*B. polymyxa* L_1_-9	the mud of the intertidal mudflat (Lianyungang Port, China)	*S. sclerotiorum* (11.0 ± 0.11 mm), *B. sorokiniana* (10.4 ± 0.21 mm), *R. solani* (9.8 ± 0.12 mm), *A. solani* (8.4 ± 0.14 mm), *B. maydis* (7.2 ± 0.06 mm) (inhibition zone diameter)	[74]

^a^ Only the most competitive values are listed in this table due to limited space.

**Table 3:** The cytotoxic activities of the secondary metabolites from marine-derived *Bacillus*.

NO.	Classification	Producing Strain	Environmental Source	Cytotoxic Activities ^a^	Ref.
**12**	Cyclic lipopeptide	*Streptomyces* sp. and *Bacillus* sp.	the mud (the intertidal mudflat in Wando, Korea)	SK-HEP-1 (27 μM), HCT116 (28 μM), MDA-MB-231 (28 μM), A549 (38 μM), SNU638 (39 μM) (IC_50_)	[25]
**28**	Linear lipopeptide	*B. subtilis* 109GGC020	sediments (the Gageocho reef, Korea)	MDA-MB-231 (14.9 µg/mL), HCT-15 (11.4 µg/mL), PC-3 (10.8 µg/mL), NCI-H23 (11.2 µg/mL), NUGC-3 (11.8 µg/mL), ACHN (11.5 µg/mL) (GI_50_)	[35]
**29**	Linear lipopeptide	*B. subtilis* 109GGC020	sediments (the Gageocho reef, Korea)	MDA-MB-231 (16.1 µg/mL), HCT-15 (18.3 µg/mL), PC-3 (19.4 µg/mL), NCI-H23 (11.7 µg/mL), NUGC-3(13.9 µg/mL), ACHN (18.4 µg/mL) (GI_50_)	[35]
**30**	Linear lipopeptide	*B. subtilis* 109GGC020	sediments (the Gageocho reef, Korea)	MDA-MB-231 (11.2 µg/mL), HCT-15 (23.2 µg/mL), PC-3 (11.7 µg/mL), NCI-H23 (10.9 µg/mL), NUGC-3 (10.5 µg/mL), ACHN (12.3 µg/mL) (GI_50_)	[35]
**45**	Nonribosomal peptide	*Bacillus* sp. PKU-MA00092	sponges, corals and sediments (the South China Sea and the southern Coast of China)	MCF7 (4.2 ± 0.1 μM), HepG2 (8.2 ± 0.2 μM) (IC_50_)	[60]
**46**	Nonribosomal peptide	*Bacillus* sp. PKU-MA00093	sponges, corals and sediments (the South China Sea and the southern Coast of China)	MCF7 (2.9 ± 0.1 μM), HepG2 (5.1 ± 0.2 μM) (IC_50_)	[60]
**47**	Nonribosomal peptide	*Bacillus* sp. PKU-MA00092	sponges, corals and sediments (the South China Sea and the southern Coast of China)	MCF7 (3.3 ± 0.1 μM), HepG2 (4.9 ± 0.2 μM) (IC_50_)	[60]
**48**	Nonribosomal peptide	*Bacillus* sp. PKU-MA00092	sponges, corals and sediments (the South China Sea and the southern Coast of China)	MCF7 (7.2 ± 0.2 μM) (IC_50_)	[60]
**84**	Other compounds	*Bacillus* sp. (SCO-147)	sediments (Suncheon Bay of Korea)	inhibitory activity (human melanoma cell line MNT-1 and melanin synthesis in mouse melanoma cell line B16F10)	[72]
**85**	Other compounds	*Bacillus* sp. (SCO-147)	sediments (Suncheon Bay of Korea)	inhibitory activity (human melanoma cell line MNT-1 and melanin synthesis in mouse melanoma cell line B16F10), enzyme inhibitory activity, and anti-pigmentary activity	[72]

^a^ Only the most competitive values are listed in this table due to limited space.

## Data Availability

Not applicable.
